# Fabry disease in South and Central Asia: Is it truly a rare disease or underappreciated?

**DOI:** 10.12669/pjms.38.8.7064

**Published:** 2022

**Authors:** Sahrai Saeed, Massimo Imazio

**Affiliations:** 1 Sahrai Saeed, Department of Heart Disease, Haukeland University Hospital, Bergenm Norway.; 2 Massimo Imazio, Cardiology, Cardiothoracic Department, University Hospital “Santa Maria della Misericordia”, ASUFC, Udine, Italy.

**Keywords:** Fabry disease, Cardiomyopathy, Enzyme replacement therapy, Incidence, South and Central Asia

## Abstract

Fabry disease (FD) is a relatively rare X-linked hereditary disease caused by mutations in the GLA gene that results in deficient α-galactosidase A (α-Gal A) enzyme activity. The disturbed catabolism of the neutral sphingolipids globotriaosylceramide (Gb3) leads to its progressive lysosomal accumulation throughout the body. Multiple organs can be affected. The atypical late-onset cardiac variant is associated with a high burden of cardiac morbidity and mortality. The true burden of FD in Central and some South Asian countries is unknown. Lack of research studies and awareness, and misdiagnosis/underdiagnosis may be the reasons. Some possible explanations as well suggestions for a structured Fabry care and research possibilities in these WHO regions are offered.

## INTRODUCTION

Fabry disease (FD) is a rare X-linked hereditary, lysosomal storage disease caused by mutations in the GLA gene (located on the X chromosome). These cause deficiency of the lysosomal enzyme alpha-galactosidase A (α-Gal A). The deficient α-Gal A activity leads to lysosomal deposition of globotriaosylceramide (GB3) and its derivatives in different cells of the body.^1^-^3^ Multiple organs can be affected. The heart, brain/central nervous system and kidneys are most commonly affected in the late-onset forms. The incidence of FD was reported to range from 1 in 40000 to 1 in 117000 live male births.^4^ This estimate may be low as screening in newborns suggests a much higher prevalence of up to 1 in 8800 newborns.^5^

### Fabry disease in Central and South Asian countries:

Overall, there are very few published reports on FD from Central and South Asian countries, except for India. The bibliographic databases PubMed, Embase was searched in August 2022 for potentially eligible articles in English using a combination of the following terms: Country name, Fabry disease, Angiokeratoma and Cardiomyopathy. Review articles, original studies and case reports/series were reviewed. By using terms “India” and “Fabry disease”, a total of 37 publications were identified. These included both review articles, original studies, case reports and series and expert opinion. Therefore, India, in line with some East-Asian countries, in whom FD is to some extent investigated, were not included in the current work. Regarding Iran, a literature search was conducted using the terms “Iran”, Fabry disease” or “Angiokeratoma”, yielding a total of seven publications with Iranian authors; two review articles^6^,^7^, an editorial^8^, two case reports of two cases each^9^,^10^, and two other publications based upon larger collaborations but without contributing local data.

There were no published reports of original data on FD from Bangladesh, Pakistan, Afghanistan, Tajikistan, Uzbekistan, Turkmenistan and Kyrgyzstan. In fact, the first two identified cases from Central Asia were from Kazakhstan which were published in *frontiers in Genetics* last year^11^; one hemizygous male and another heterozygous female. Both presented in adulthood with a delayed diagnosis. The authors conducted a thorough familial screening which led to the identification of further 10 affected family members (eight females).

In Pakistan, except for two review articles, one on FD published in *Journal of the Pakistan Medical Association* in 2014^12^ and another article on overall lysosomal storage diseases in a paediatric population, published in *Pakistan Journal of Medical Sciences* in 2017^13^, and a series of 14 cases of angiokeratoma of tongue, published in *Journal of the Pakistan Medical Association* in 2006^14^, there were no other original articles on FD. Of note, in both review articles^12^-^13^, no local studies from Pakistan or from the region were reported. Similarly, no review article or original data have been published by the two other leading medical journals in the country: *Pakistan Journal of Medical Sciences* and *JCPSP-Journal of the College of Physicians and Surgeons Pakistan*. Hence, in view of these considerations two important questions arise:

1) Is FD truly a rare disease in these WHO regions; or
2) Is it underappreciated because of underdiagnosis/misdiagnosis or lack of awareness?

The latter is probably true. However, in the absences of original studies, it may be difficult to address these uncertainties. Nevertheless, some possible explanations can be offered:

1) FD may be underdiagnosed or misdiagnosed due to lack of awareness;
2) The true incidence may be lower than that in European populations;
3) FD may not be the research focus, probably due to low number of patients and lack of nationwide registries and research funding to conduct larger collaborative studies.

In order to increase awareness about FD and stimulate Fabry research, the following initiatives may be needed:

1. Establish local guidelines on management of FD in children and adults.
2. Screening of families and groups at risk to improve diagnostic process. Particularly, pedigree analysis helps to establish which relatives of Fabry patients are at risk of the disease, enabling a more effective treatment for underdiagnosed or misdiagnosed individuals.
3. Setting out nationwide registries and establishing specialist Fabry centers in tertiary hospitals, interdisciplinary teams, and regional competence centers. Because patient registries provide long-term, real-world evidence that is essential for the understanding of the natural history, disease progression, monitoring the effects of treatment on a large patient population with rare diseases**.** This can be exemplified by FOS (Fabry Outcome Survey), an international, multicentre, observational registry (NCT03289065) which helped documenting the natural history of FD and provided significant amount of clinical data and analyses to support improvement in patient management.^15^
4. Further develop and validate blood spot methodology for the measurement of alpha galactosidase enzyme. Early establishing of diagnosis is essential as misdiagnosis leads to delay in identifying FD, particularly in the case of heterogenous clinical manifestations.
5. Patient organizations to provide support and information.
6. Most importantly providing financial support and free care including ERT – a high-cost treatment which can only be provided by the social security system, particularly in countries with high poverty burden.

These initiatives will hopefully provide a better answer on the true incidence, phenotypes of presentation, burden of cardiac disease in the late-onset variant, in above mentioned WHO regions.

## CONCLUSIONS

The true burden of FD in Central and some South Asian countries is unknown, and lack of awareness and underdiagnosis/misdiagnosis may be the reasons. Building nationwide registries, specialist Fabry clinics and research collaborations provide long-term, real-world data, which is essential for understanding the natural history of FD as well as monitoring the effects of treatment.

### Note:

The opinion expressed in the present expert commentary is the view of the authors and does not necessarily reflect the view of the institutions the authors belong to.

### Author Contributions:

**SS** wrote the first draft of the article which was revised by **MI**.

Both authors approved the final submission.
